# Inoculated mammary carcinoma-associated fibroblasts: contribution to hormone independent tumor growth

**DOI:** 10.1186/1471-2407-10-293

**Published:** 2010-06-16

**Authors:** Victoria T Fabris, Ana Sahores, Silvia I Vanzulli, Lucas Colombo, Alfredo A Molinolo, Claudia Lanari, Caroline A Lamb

**Affiliations:** 1 Laboratory of Hormonal Carcinogenesis, Instituto de Biología y Medicina Experimental (Consejo Nacional de Investigaciones Científicas y Técnicas - CONICET), Vuelta de Obligado 2490, Buenos Aires, Argentina; 2 Instituto de Investigaciones Oncológicas, Academia Nacional de Medicina, Las Heras 3092, Buenos Aires 1425, Argentina; 3 Area Investigación, Instituto de Oncología AH Roffo, Universidad de Buenos Aires, Buenos Aires, Argentina; 4 Oral and Pharyngeal Cancer Branch, National Institute of Craniofacial and Dental Research, NIH, Bethesda, MD 20892-4340, USA

## Abstract

**Background:**

Increasing evidence has underscored the role of carcinoma associated fibroblasts (CAF) in tumor growth. However, there are controversial data regarding the persistence of inoculated CAF within the tumors. We have developed a model in which murine metastatic ductal mammary carcinomas expressing estrogen and progesterone receptors transit through different stages of hormone dependency. Hormone dependent (HD) tumors grow only in the presence of progestins, whereas hormone independent (HI) variants grow without hormone supply. We demonstrated previously that CAF from HI tumors (CAF-HI) express high levels of FGF-2 and that FGF-2 induced HD tumor growth *in vivo*. Our main goal was to investigate whether inoculated CAF-HI combined with purified epithelial (EPI) HD cells can induce HD tumor growth.

**Methods:**

Purified EPI cells of HD and HI tumors were inoculated alone, or together with CAF-HI, into female BALB/c mice and tumor growth was evaluated. In another set of experiments, purified EPI-HI alone or combined with CAF-HI or CAF-HI-GFP were inoculated into BALB/c or BALB/c-GFP mice. We assessed whether inoculated CAF-HI persisted within the tumors by analyzing inoculated or host CAF in frozen sections of tumors growing in BALB/c or BALB/c-GFP mice. The same model was used to evaluate early stages of tumor development and animals were euthanized at 2, 7, 12 and 17 days after EPI-HI or EPI-HI+CAF-HI inoculation. In angiogenesis studies, tumor vessels were quantified 5 days after intradermal inoculation.

**Results:**

We found that admixed CAF-HI failed to induce epithelial HD tumor growth, but instead, enhanced HI tumor growth (p < 0.001). Moreover, inoculated CAF-HI did not persist within the tumors. Immunofluorescence studies showed that inoculated CAF-HI disappeared after 13 days. We studied the mechanisms by which CAF-HI increased HI tumor growth, and found a significant increase in angiogenesis (p < 0.05) in the co-injected mice at early time points.

**Conclusions:**

Inoculated CAF-HI do not persist within the tumor mass although they play a role during the first stages of tumor formation promoting angiogenesis. This angiogenic environment is unable to replace the hormone requirement of HD tumors that still need the hormone to recruit the stroma from the host.

## Background

Breast cancers are comprised of epithelial neoplastic cells embedded in a tumor microenvironment composed mainly of extracellular matrix and non-neoplastic cells such as inflammatory cells, vascular cells and fibroblasts [1]. Fibroblasts growing within the tumor mass are known as carcinoma-associated fibroblasts (CAF). They express smooth muscle actin and are found in large numbers in most invasive human breast cancers [2]. There is increasing evidence that CAF play different roles supporting tumor growth [3]. Several studies have addressed the role of CAF using experimental designs that involve the co-inoculation of CAF with breast cancer cell lines in mice [4,5]. However, the persistence of the inoculated CAF within the carcinoma remains controversial. Some authors have reported that they have detected the inoculated CAF for periods of 30 days or more after the injection [5,6], suggesting that the tumor stroma was derived from the inoculated CAF, while others have reported that the stroma within the tumor was derived from the host [4,7,8].

In our laboratory, we work with the MPA mouse model of mammary carcinomas. These tumors were induced by the continuous administration of medroxyprogesterone acetate (MPA) in BALB/c mice. Most tumors are initially hormone dependent (HD) because they grow only if MPA is supplied. However, they can give rise to different hormone-independent (HI) variants, which are able to grow without hormone supply. Both HD and HI tumors, have a rich stromal compartment [9-11]. We have reported that isolated CAF from our model can be stimulated by EGF, TGFβ1 and FGF-2 [12], but they are not stimulated by steroid hormones. In previous studies, we also demonstrated that primary cultures of isolated CAF from HI tumors (CAF-HI) induced an increase in cell proliferation of epithelial cells isolated from HD tumors (EPI-HD). These CAF-HI express higher levels of fibroblast growth factor 2 (FGF-2) than CAF from HD tumors (CAF-HD), and the exogenously administrated FGF-2 induced in vivo tumor growth of HD tumors in the absence of MPA [13].

In this study, we wanted to reproduce these in vitro findings in an in vivo setting and we hypothesized that CAF-HI, by secreting FGF-2 or other paracrine factors, would be able to promote the growth of HD tumor cells. Our main goal was to evaluate the effect of co-inoculated CAF-HI on tumor growth of EPI-HD and EPI-HI cells. This report clearly demonstrates that inoculated CAF-HI do not persist within the tumor mass although they may participate during the first stages of tumor growth, favoring angiogenesis. This angiogenic milieu increases HI tumor growth, although this stimuli is not sufficient to replace the hormone requirement of HD tumors.

## Methods

### Animals

Two-month-old virgin female BALB/c mice (Animal Facility, Instituto de Biología y Medicina Experimental) were housed in groups of four per cage. Transgenic mice expressing enhanced GFP under the direction of the human ubiquitin C promoter were purchased from the Jackson Laboratories, Bar Harbor, Maine. These mice express Green Fluorescent Protein (GFP) in all tissues examined. The animals were in an air-conditioned room at 20 ± 2°C with a 12-hour light/dark cycle and had access to food and water ad libitum. Animal care and manipulation were in agreement with institutional guidelines and the Guide for the Care and Use of Laboratory Animals [14]. Protocols are approved by the Institutional Bioethical Committee.

### Tumors

C4-HD, a transplantable ductal mammary tumor, was originally induced by the continuous administration of MPA to a BALB/c female mouse [9,15] and was subsequently maintained by serial subcutaneous (sc) transplantations into syngeneic MPA-treated female mice. This HD tumor expresses high levels of estrogen (ER) and progesterone receptors (PR) [16] and shows a nearly diploid karyotype [17]. The C4-HI variant arose from the C4-HD tumors when it started to grow in the absence of MPA. C4-HI cells also exhibit high ER and PR levels but have acquired an aneuploid karyotype [17,18].

### Primary cultures

Primary cultures were prepared from C4-HI and C4-HD tumors as described previously [16,19]. Briefly, tumors were aseptically removed, minced, washed with DMEM/F12 medium (Dulbecco's modified Eagle's medium: Ham's F12, 1:1, without phenol red, Sigma Chem. Co.), suspended in 5 ml of enzymatic solution [2.5 mg/ml trypsin (Life Technologies Inc.), 5 mg/ml albumin (Life Technologies Inc.), and 850 U/ml collagenase type II (Life Technologies Inc.) in phosphate buffered saline], and incubated at 37°C for 20 minutes with continuous stirring. The liquid phase of the suspension was then removed, and the undigested tissue was incubated for an additional 20 minutes with fresh enzymatic solution. Enzyme action was interrupted by adding a washing medium with 10% fetal calf serum (FCS, BioSer, Buenos Aires, Argentina). Epithelial (EPI) and fibroblastic cells (CAF) were isolated by resuspending the digested material in 20 ml of medium plus 10% FCS and allowing it to settle for 20 minutes. The upper 7 ml, containing single cells, were seeded into plates and the medium was changed after two hours. This time period is enough for the CAF to settle and not enough for the EPI to attach to the plastic plates. The fraction corresponding to the lower 5 ml, containing the sedimented cells, was resuspended in another 20 ml of washing medium (plus 5% FCS) and allowed to settle for 20 minutes. The upper 15 ml were discarded and the procedure was repeated 10 times, or until no single cells were observed in the upper fraction. Instead of plating the purified EPI, they were directly used for in vivo experiments. CAF, on the other hand, were obtained from primary cultures that were grown for one week prior to inoculation.

### Immunofluorescence

Purified EPI-HI, CAF-HI or EPI-HI+CAF-HI were seeded onto chamber slides. Slides were fixed with 100% ethanol for 30 minutes at -20°C, washed and treated with 0.25% Triton X-100 for 20 minutes. Slides were rinsed and blocked with 5% FCS for one hour at room temperature. Incubation with cytokeratin (CK; polyclonal rabbit antibody Z0622, 1:250 dilution, DakoCytomation, Carpinteria, CA) or smooth muscle actin (SMA; monoclonal mouse antibody Sigma Aldrich) was performed overnight at 4°C. After rinsing in PBS, slides were incubated with fluorescein isothiocyanate (FITC)-conjugated anti-rabbit (for CK) or anti-mouse (for SMA) IgG at 1:100 dilution (Vector Laboratories, Burlingame, CA). Nuclei were counterstained with propidium iodide (PI). The signal was viewed in a Nikon Eclipse E800 confocal microscope.

### In vivo experiments

### Tumor growth

In the first set of experiments, untreated female BALB/c mice were sc inoculated with 1.5 × 10^4 ^EPI-HD or EPI-HI alone or together with the same number of CAF-HI (n = 6 mice/group). Isolated CAF-HI were also inoculated to rule out the possibility that contaminating EPI-HI cells could account for the observed effects. In the second set of experiments, 1.5 × 10^4 ^EPI-HI or EPI-HI+CAF-HI were sc inoculated in untreated female BALB/c mice and the animals were euthanized at 2, 7, 12 and 17 days post-inocula (n = 3/group). These cell injections were mixed with trypan blue prior to inoculation to localize the area of inoculation. After removal, isografts were immediately fixed in 10% formalin, embedded in paraffin and prepared for histological evaluation.

### Tracking inoculated CAF-HI

#### Female CAF-HI in male BALB/c mice

EPI-HI (1.5 × 10^4^) alone or together with an equal number of CAF-HI were sc inoculated into female (n = 4-6/group) or male untreated BALB/c mice (n = 4/group). Animals were followed closely, and tumor width and length were measured 3 times a week using a Vernier Caliper. Animals were euthanized after one month. Some of the tumors were frozen for in situ hybridization studies.

#### GFP-labeled CAF-HI in BALB/c mice

EPI-HI (1.5 × 10^4^) alone or together with an equal amount of CAF-HI-GFP were sc inoculated in BALB/c mice. Trypan blue was used to visualize the site of injection.

#### Unlabeled CAF-HI in BALB/c-GFP mice

EPI-HI (1.5 × 10^4^) alone or together with an equal amount of unlabeled CAF-HI were sc inoculated in BALB/c-GFP. Trypan blue was used to visualize the site of injection.

#### Tumor transplant growing in BALB/c-GFP mice transplanted in BALB/c mice

EPI-HI were sc inoculated into BALB/c-GFP mice. Once the tumor grew a small piece was transplanted into BALB/c mice.

For all in vivo experiments performed with BALB/c-GFP mice or inoculated CAF-HI-GFP, before the tumors were excised, the animals were perfused with a cold saline solution (0.9% NaCl) followed by 4% paraformaldehyde (PFA). The inoculated area was excised at different time points (4, 7, 13 and 28 days), kept in cold 4% PFA overnight and then transferred to 20% sucrose for another 24 hours. Tissues were embedded in OCT (Tissue-Tek O.C.T Compound), frozen sections were cut and the endogenous fluorescence was analyzed by confocal microscopy. Nuclei were counterstained with PI. Primary cultures followed by SMA immunostaining were performed with some of these tumors.

### Mast and PMN cell quantification

BALB/c mice (n = 3-10/group) were sc inoculated with saline, CAF-HI (1.5 × 10^4^), EPI-HI (1.5 × 10^4^), or EPI-HI+CAF-HI (1.5 × 10^4 ^+ 1.5 × 10^4^). Trypan blue was used to visualize the site of injection. The animals were euthanized at 7 and 12 days post inocula; tumors were then fixed with 10% formalin and embedded in paraffin.

#### Mast cells-toluidine blue method

Tumor sections were deparaffinized and rehydrated, stained for 30 minutes in aqueous toluidine blue (0.1% pH 1), washed and nuclei were counterstained with methyl green. Tissues were dehydrated and mounted on Permount and observed under a microscope. Mast cells were identified by their characteristic metachromasia. Total mast cells within the inoculated area were counted using a Nikon Eclipse E200 microscope, magnification 400×. The tumor area was measured using "Image J" Software and the numbers of mast cells were calculated per area.

#### Polymorphonuclear cells quantification

Hematoxylin and eosin (H&E) staining was observed under a Nikon Eclipse E200 microscope. PMN cells were identified by standard morphological criteria (Magnification: 1000×). All fields within the inoculated area were counted and PMN numbers of cells were referred per field.

### Fluorescence in situ hybridization (FISH) and immunofluorescence

Frozen tissue sections of the tumors were fixed in a cold solution of methanol:acetic acid (3:1) for one hour. The tissue sections were denatured in 70% formamide/2× standard saline-citrate. Mouse paint biotinylated DNA probes (Cambio, Cambridge, UK) for chromosomes X and Y were denatured and preannealed. The hybridization was performed overnight at 37°C. After hybridization, the slides were washed and the probes were detected with Avidin-TexasRed (Vector Laboratories, Burlingame, CA). Immunofluorescence using a CK antibody (polyclonal rabbit antibody Z0622, 1:250 dilution, DakoCytomation, Carpinteria, CA) was performed on the same slides. A secondary anti-rabbit-FITC coupled antibody was used. Nuclei were counterstained with DAPI. The signal was viewed in a Nikon Eclipse E800 confocal microscope.

### Angiogenesis assay in vivo

Each BALB/c mouse was implanted intradermally on the ventral surface with the cells. Saline, CAF-HI (1.5 × 10^4^), EPI-HI (1.5 × 10^4^) or EPI-HI+CAF-HI (1.5 × 10^4 ^+ 1.5 × 10^4^) were injected in a volume of 50 μl together with one drop of 0.4% trypan blue to visualize the sites of injection. After 5 days, the mice were sacrificed, the skin carefully separated, and photographs captured under a Trinocular Zoom Stereomicroscope (Leica). The number of vessels was scored using a grid, subdivided into squares of 0.16 mm^2^, and superimposed onto the photograph of the injection site.

### Statistical Analysis

Tumor growth curves were studied using regression analysis and the slopes were compared using ANOVA followed by parallelism analysis. Unpaired *t-*tests were used as needed. Values are considered significant if p < 0.05.

## Results

### Effect of CAF-HI on HD and HI tumor growth in vivo

**Figure 1:**
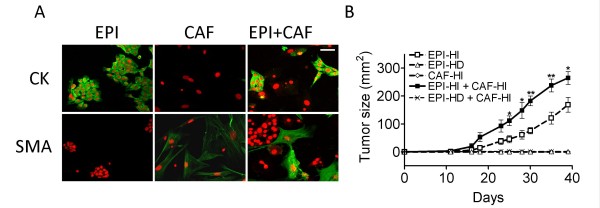
**Purified EPI and/or CAF inoculated in vivo**. (A) Immunofluorescence of purified EPI-HI, CAF-HI or EPI-HI+CAF-HI. (Upper panel) Cytokeratin staining (CK; Rabbit polyclonal antibody Dako). (Lower panel) SMA (Mouse monoclonal antibody, Sigma Aldrich). Anti-rabbit and anti-mouse secondary antibodies coupled to FITC (Vector Laboratories) were used for CK and SMA staining, respectively (green). Cells were seeded on chamber slides and fixed in 100% ethanol for 30 minutes at -20°C. Immunofluorescence was performed as described in Materials and Methods. Nuclei were counterstained with PI (red). Original magnification: 400×. Bar: 60 μm. (B) Tumor growth of purified EPI-HD or EPI-HI cells and CAF-HI (2 × 10^5 ^cells) alone or EPI (2 × 10^5 ^cells) plus CAF-HI (2 × 10^5^) that was implanted sc in female BALB/c mice (n = 6). EPI-HI or EPI-HI+CAF-HI were prepared as described in Materials and Methods. **: p < 0.01; *: p < 0.05. No tumor growth was observed in EPI-HD, CAF-HI or EPI-HD+CAF-HI. Tumor size: Mean ± s.e.m.

Primary cultures from HD and HI tumors were generated, and the epithelial cells (EPI) and CAF were separated as described in Materials and Methods. We verified the purity of both cell populations by immunostaining. Isolated EPI strongly expressed CK whereas CAF expressed the fibroblastic marker SMA (Figure 1A) and vimentin (data not shown). This method proved to be very effective for the isolation of EPI. As confirmed by CK staining, a purity of 99 ± 0.5% could be attained. CAF enriched cultures were 93 ± 9.8% pure. To assess the contribution of CAF-HI to HD tumor growth, we inoculated these cell populations either alone or in combination. In other words, we mixed CAF-HI with EPI-HD or EPI-HI cells in a 1:1 ratio and inoculated these mixtures sc in BALB/c mice. We also inoculated EPI-HD, EPI-HI and CAF-HI alone. EPI-HI co-mixed with CAF-HI induced a significant increase in tumor size at the end of the experiment (Figure 1B), a decrease in tumor take (p < 0.05; EPI-HI vs. EPI-HI+CAF-HI) and an increase in tumor growth rate during the first period of tumor development (Slope EPI-HI: 5.2 ± 0.5 mm^2^/day; EPI-HI + CAF-HI: 8.3 ± 0.5 mm^2^/day p < 0.01). To ensure that contaminating EPI cells could not account for the enhancement of EPI-HI tumor growth, 2 × 10^5 ^and 2.2 × 10^5 ^cells (plus 10% EPI-HI) were inoculated. No differences were observed between either group (data not shown), indicating that the inoculated CAF-HI were responsible for promoting tumor growth. Moreover, no tumor growth was observed in EPI-HD co-inoculated with CAF-HI. These results raised two possibilities: CAF-HI do not provide sufficient stimuli to induce EPI-HD tumor growth, or inoculated CAF-HI do not remain in the tumor for initial signaling to induce EPI-HD tumor growth and thus, to bypass the hormone requirement.

### Localization of inoculated CAF-HI at different time points

**Figure 2:**
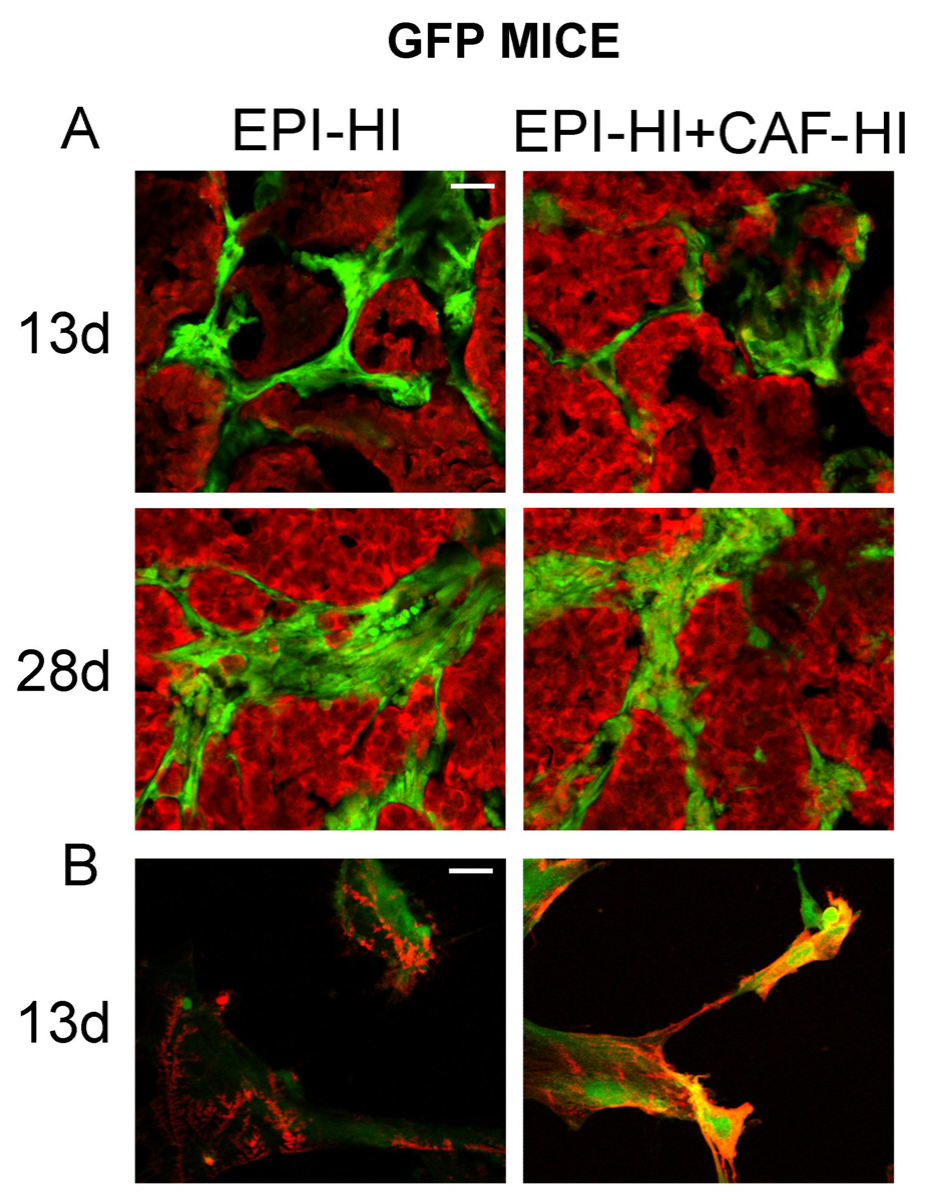
**Cryostat sections of isolated EPI-HI or admixed with CAF-HI growing in BALB/c-GFP mice**. (Upper panel) EPI-HI alone or admixed with CAF-HI were sc inoculated and animals were sacrificed at 13 or 28 days post inoculation. Before sacrifice, animals were perfused with 4% PFA. Cryostat sections were prepared; nuclei were counterstained with PI and sections visualized in a confocal microscope. Original magnification: 200×. Bar: 60 μm. (Lower panel) Immunofluorescence for SMA of primary cultures of EPI-HI and EPI-HI+CAF-HI tumors on day 13 after inoculation. Cells were seeded in chamber slides, fixed with 10% formalin and immunofluorescence was performed as described in Materials and Methods. Nuclei were counterstained with PI and slides were visualized in a confocal microscope. Original magnification: 400×. Bar: 60 μm.

To address the possibility that inoculated CAF-HI do not persist in the tumor and that the host stroma forms part of the tumor mass, we developed a murine isograft model that enabled us to distinguish the cells that come from the host from those that come from the donor. Thus, we isolated EPI-HI and CAF-HI from tumors growing in BALB/c mice and inoculated the neoplastic cells alone or admixed with CAF-HI in BALB/c-GFP mice. These mice express the GFP in all tissues examined (Jackson lab UBC-GFP). As shown in Figure 2A by fluorescent analysis of frozen sections, as early as 13 days after cell injection, there is already a strong invasion of host stroma within the tumor mass even in the admixed group. Primary cultures of these tumors and using an anti-smooth muscle actin antibody confirmed that all the SMA positive cells were positive for GFP too (Figure 2B), suggesting that even at early time points after cell inoculation the inoculated CAF-HI are undetected. These data were supported by additional assays in which neoplastic female cells were inoculated alone or admixed with CAF-HI in male BALB/c mice and the results were analyzed by FISH (data not shown).

**Figure 3:**
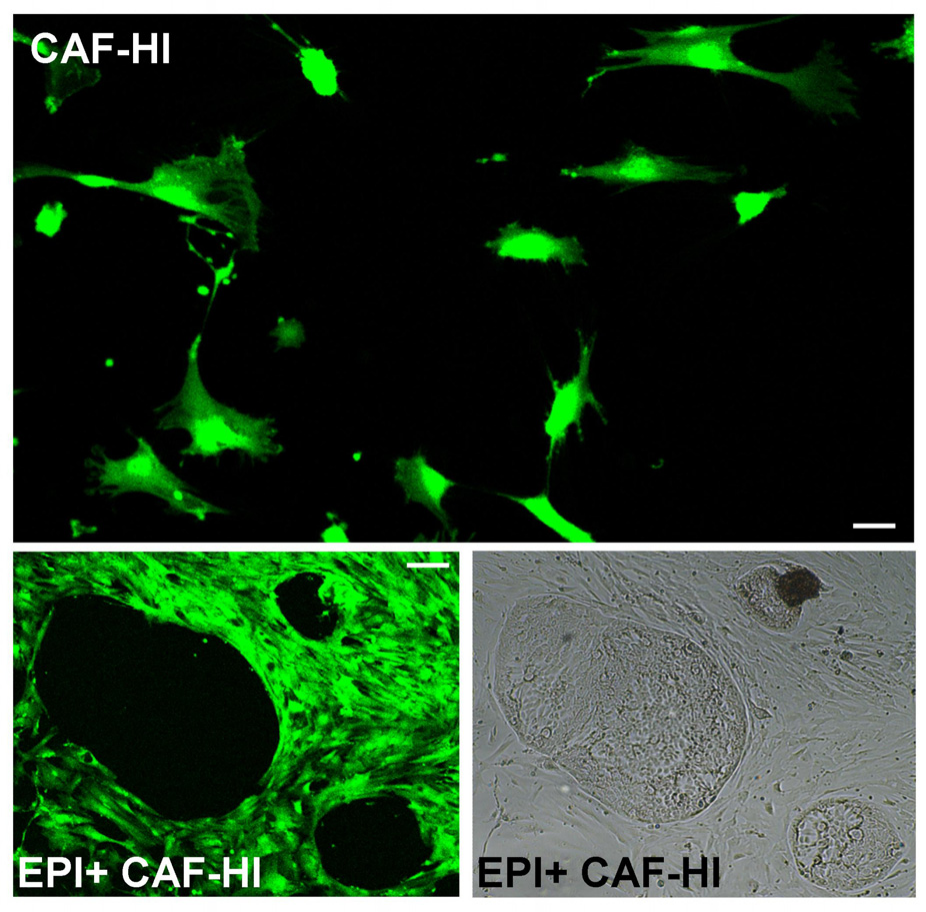
**Primary cultures of C4-HI tumors growing in BALB/c-GFP mice**. CAF-HI-GFP were isolated as described in Materials and Methods. (Upper panel) Photograph of a primary culture of isolated CAF-HI-GFP alone or, (lower panel) combined with EPI-HI cells. Notice that epithelial neoplastic cells are negative for GFP. Original magnification: 100×; Bar: 30 μm. (upper panel) and 40× Bar: 75 μm (lower panel).

**Figure 4:**
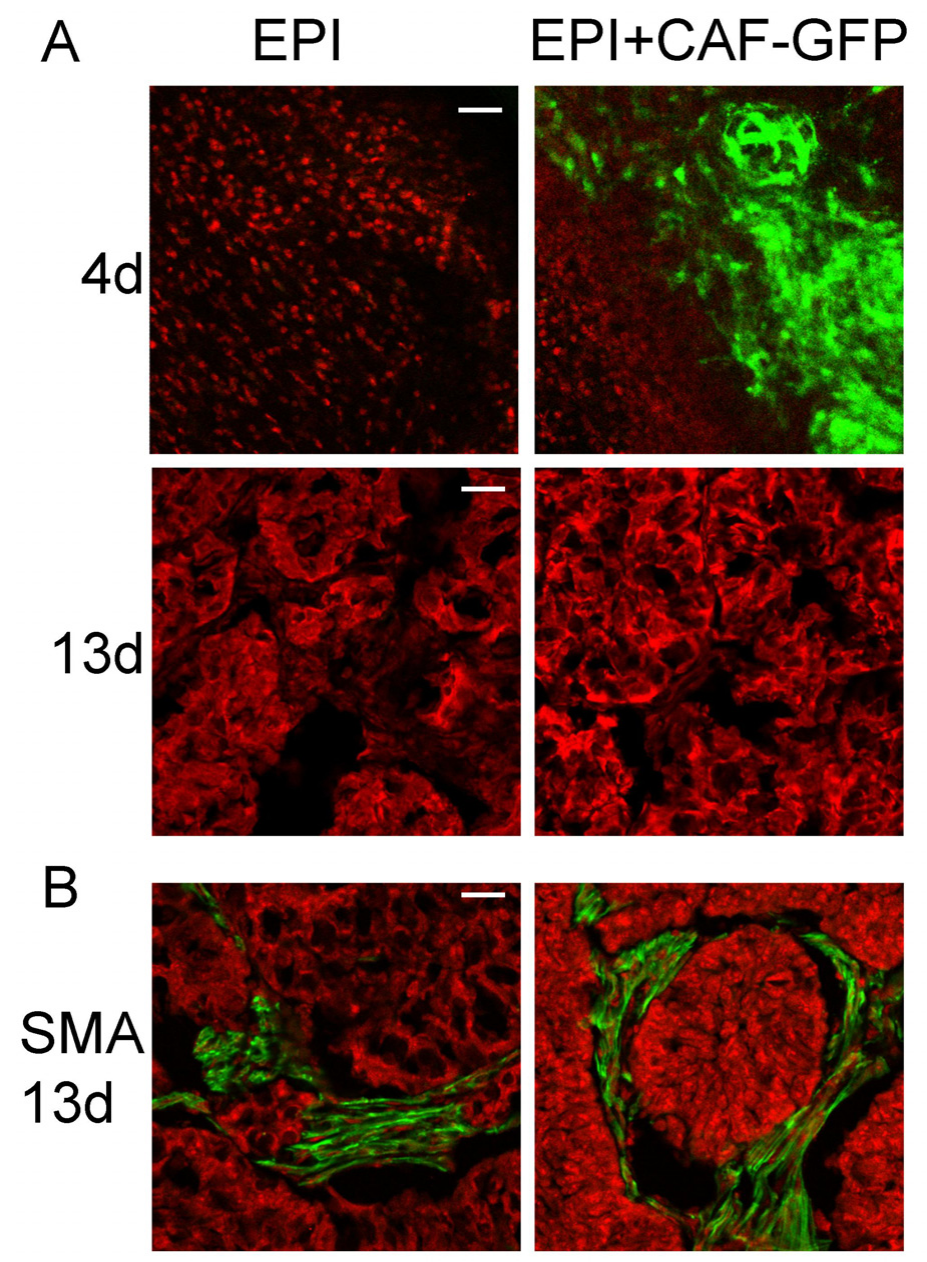
**Cryostat sections of isolated EPI-HI or admixed with CAF-HI-GFP growing in BALB/c mice**. (Upper panel) EPI-HI alone or admixed with CAF-HI-GFP were sc inoculated and mice were sacrificed at 4 or 13 days post inoculation. Before sacrifice, animals were perfused with 4% PFA. Cryostat sections were prepared, nuclei were counterstained with PI, and sections were visualized in a confocal microscope. Original magnification: 100× (Day 4) Bar: 30 μm; 200× (Day 13) Bar: 60 μm. (Lower panel) Immunofluorescence for SMA on day 13 after injection for EPI-HI and EPI-HI+CAF-HI tumors. Nuclei were counterstained with PI, and the sections were visualized in a confocal microscope. Original magnification: 200× (Day 13) Bar: 60 μm.

**Figure 5:**
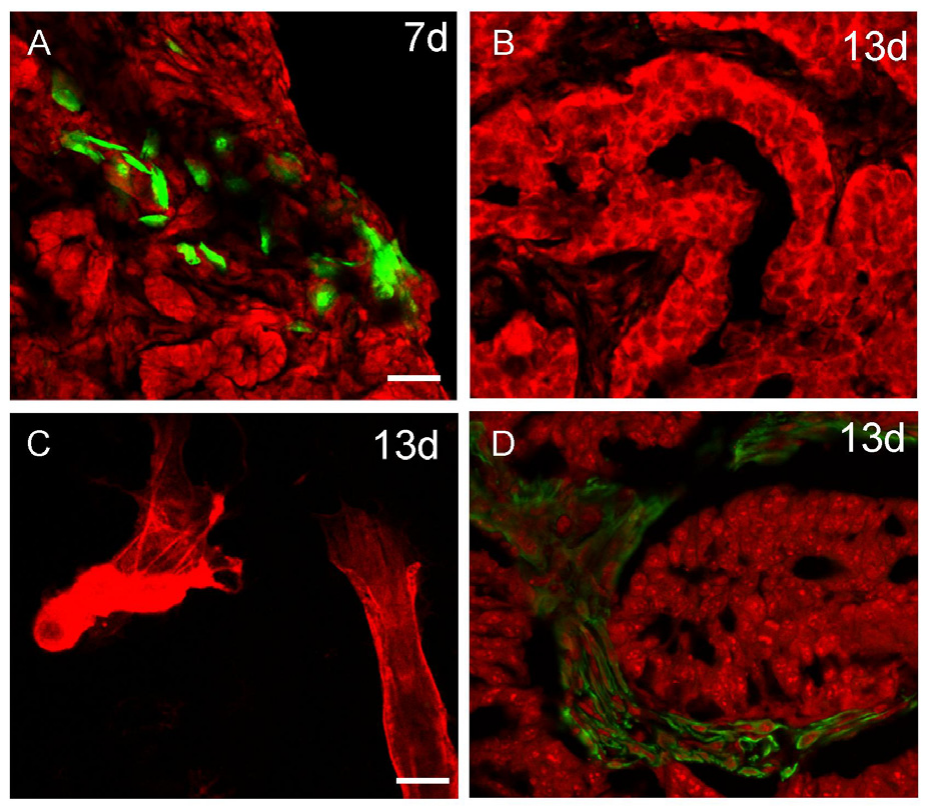
**Transplant of C4-HI tumor growing in BALB/c-GFP mice into BALB/c mice**. Mice were sacrificed 7 days (A) or 13 days (B-D) after transplantation. Before sacrifice animals were perfused as described in Materials and Methods and cryostat sections were obtained. (A-B) Endogenous GFP labeling was detected under a confocal microscope. (C) Primary cultures of 13-day tumors were seeded on chamber slides and CAF-HI were immunostained for SMA (red). Original magnification: 400×; Bar: 45 μm. (D) Cryostat sections of 13-day tumors were immunostained for SMA (green). Nuclei were counterstained with PI. Original magnification: 200×; Bar: 60 μm.

We also performed the opposite experiment. We separated CAF-HI from a 28-day HI tumor growing in BALB/c-GFP mice. As expected, these CAF-HI expressed high levels of GFP (Figure 3). We then inoculated them with EPI-HI into BALB/c mice. Inoculated CAF-HI-GFP were not detected 13 days after transplantation (Figure 4A). However, the non-fluorescent tumor stroma was positively stained with SMA in immunofluorescence studies, demonstrating that the inoculated CAF-HI do not persist within the tumor and that neoplastic epithelial cells recruit new CAF from the host (Figure 4B). Furthermore, primary cultures of these tumors followed by immunofluorescence showed that 100% of SMA positive cells were negative for GFP (data not shown). In a different set of experiments, we transplanted EPI-HI into BALB/c-GFP mice and a small piece of this tumor was transplanted into BALB/c mice. Fluorescence analysis of frozen sections displayed similar results: 13 days after tumor transplantation, no GFP stromal cells were detected within the tumor mass (Figure 5A and 5B), and the non-fluorescent stroma was stained with SMA (Figure 5D). In addition, isolated CAF-HI, which were positive for SMA, were 100% negative for GFP (Figure 5C). These results indicate that even CAF within tumor transplants do not persist in the tumors after trocar transplantation and neoplastic cells recruit the stroma from the host.

### Effect of CAF-HI on histology during early stages of tumor formation

**Figure 6:**
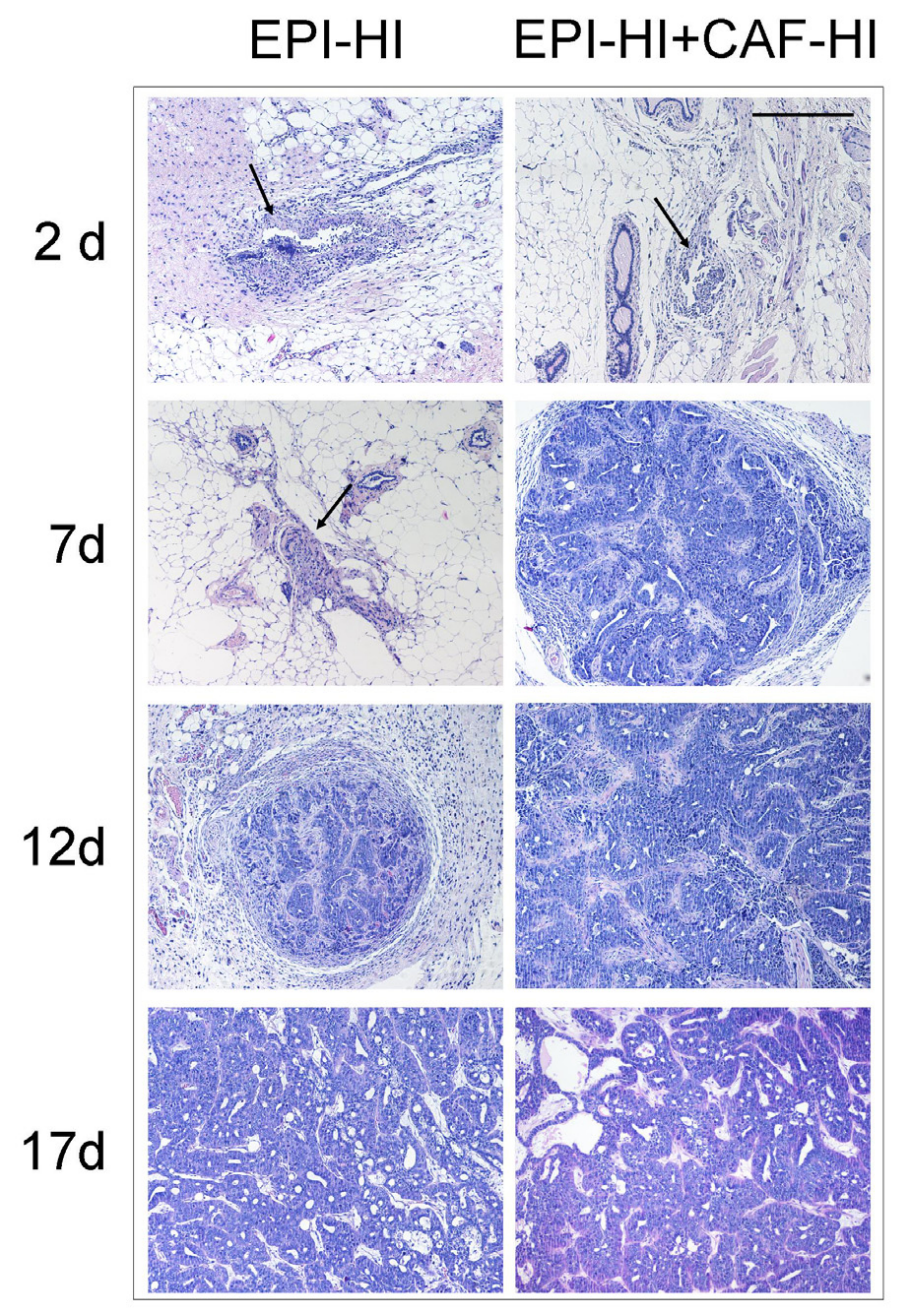
**Histological examination at early stages of tumor development of EPI-HI or EPI-HI+CAF-HI isografts**. The isografts were excised at 2, 7, 12 and 17 days after inoculation. BALB/c mice were sc inoculated with CAF-HI and/or EPI-HI cells. Original magnification: 100×. Bar: 170 μm.

We demonstrated that inoculated CAF-HI do not participate in HD tumor growth; however, they enhanced HI tumor growth (Figure 1B). Thus, our next goal was to investigate the growth-enhancing effects of CAF-HI on HI tumor growth during the first days of tumor formation. EPI-HI alone or mixed with CAF-HI were inoculated into female BALB/c mice. At 2, 7, 12 and 17 days after inoculation, the area of tumor inoculum was excised and histologically evaluated. At day 2 after cell injection, inflammatory infiltrates such as monocytes, lymphocytes and polymorphonuclear leukocytes (PMN) were present in both groups. There were no evident signs of vascularization and it was clear that the inoculated isografts had not yet developed into solid tumors (Figure 6). At day 7, significant differences were observed between both groups. The mixed isografts generated tumors with ductal and cribiform differentiation, which are the morphological features of these tumors, that were still absent in EPI-HI isografts (Figure 6). Several lymphocytes and polymorphonuclear cells were observed surrounding the tumors (Figure 6). Despite the clear differences on tumor histology observed at day 7, on day 12, the EPI-HI cells developed tumors that were very similar to the EPI-HI+CAF-HI carcinomas observed at day 7 (Figure 6). At subsequent time points, the tumors continued to grow, although the EPI-HI lesions were always smaller than the admixed tumors (p < 0.001). Even though EPI-HI isografts began growing later than EPI-HI+CAF-HI, the tumor growth rate was similar (Figure 1B). It is evident, that there are no histological differences in either tumor except for the lag in the development of the tumor structure. These results suggested that the inoculated CAF-HI influence the microenvironment during the first stages of tumor formation, contributing to early tumor development.

### Effect of inoculated CAF-HI on tumor angiogenesis

**Figure 7:**
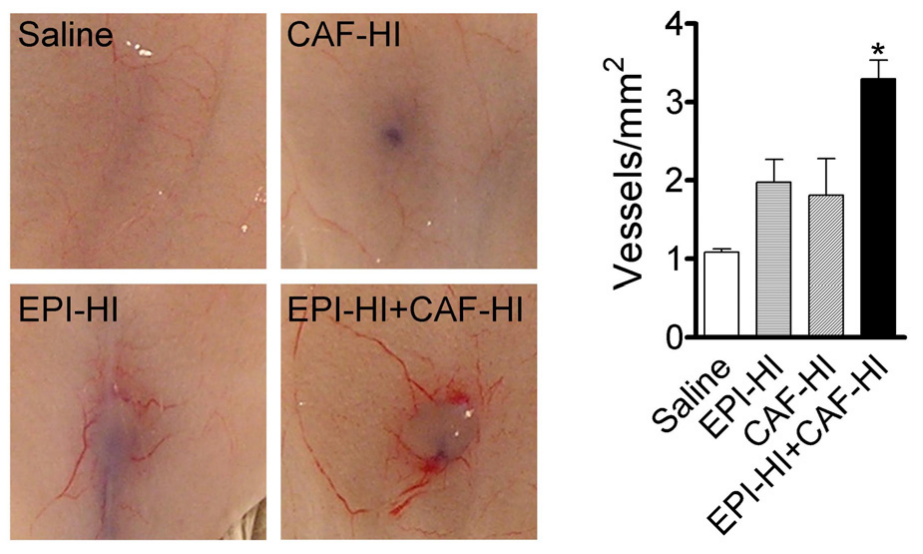
**Tumor angiogenesis at early stages of tumor development**. BALB/c mice were intradermally inoculated with saline, CAF-HI (1.5 × 10^4^), EPI-HI (1.5 × 10^4^) or EPI-HI+CAF-HI (1.5 × 10^4 ^+ 1.5 × 10^4^). Mice were sacrificed 5 days after inoculation; the site of injection was exposed and viewed under a Trinocular Zoom Stereomicroscope. The graph shows the quantification of vessels per area. Vessel density was determined using a grid that was superimposed onto the photograph at the injection site.

We previously demonstrated that FGF-2, a well known angiogenic factor [20], exerts a direct proliferative effect on epithelial HD and HI cells [13] and that CAF-HI express higher levels of FGF-2 than CAF-HD. To investigate if co-inoculated CAF-HI could influence angiogenesis during the first stages in tumor development, we performed intradermal inoculations of saline, CAF-HI, EPI-HI or EPI-HI+CAF-HI in BALB/c mice. Trypan blue stain was co-administered with the cells to localize the site of the inoculum. Mice were sacrificed 5 days after inoculation and tumor vessels were counted. A significant increase in tumor vessels (p < 0.05) was observed in co-inoculated animals compared to the other groups (Figure 7). Similarly, endothelial cells were positive for Von Willebrand factor expression in the vascular structures at the outside edge of EPI-HI+CAF-HI tumors, whereas no vessels were evident in EPI-HI isografts at this time point (data not shown). Collectively, these results indicate that inoculated CAF-HI induce angiogenesis at the site of injection contributing to rapid tumor formation.

### Effect of inoculated CAF-HI on the host immune response

**Figure 8:**
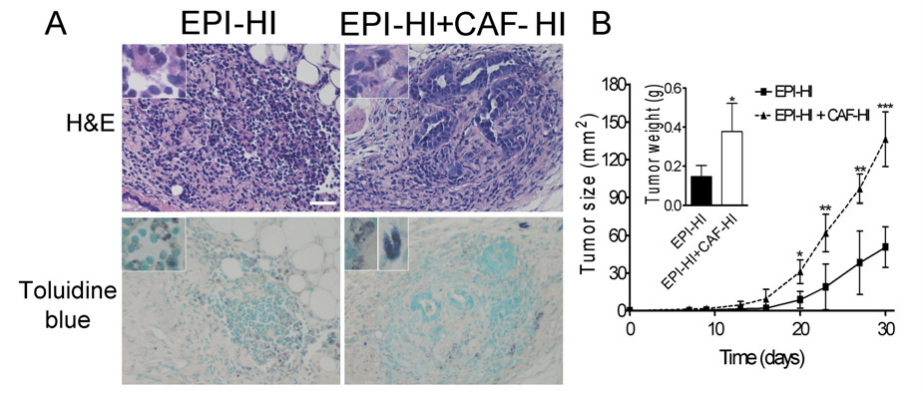
**PMN and mast cells at early stages (5 days) of tumor development**. (A) Hematoxylin and eosin (upper panel) or toluidine blue (lower panel) staining of EPI-HI or EPI-HI+CAF-HI isografts. BALB/c mice were sc inoculated with CAF-HI and/or EPI-HI cells. Original magnification: 100× Bar: 72 μm. (Insets) Magnification showing (upper panel) PMN cells or (lower panel) mast cells within the inoculation site. (B) Tumor growth of purified EPI-HI (1.5 × 10^4 ^cells) alone or admixed with CAF-HI (1.5 × 10^4^) implanted sc in NOD/SCID mice (n = 4-5). EPI-HI or EPI-HI+CAF-HI were prepared as described in Materials and Methods. ***: p < 0.001; **: p < 0.01; *: p < 0.05. Tumor size: Mean ± s.d. (Inset) Tumor weight at the end of the experiment (day 30) for EPI-HI and EPI-HI+CAF-HI tumors.

To investigate whether the inoculated CAF-HI could be modulating the innate host's immune system, we evaluated at 5 days post-inoculum the number of PMN and mast cells using standard morphological criteria and toluidine blue staining, respectively. Both cell types were detected but we found no significant differences between both groups (Figure 8A). To rule out the role of the immune system we inoculated EPI-HI alone or commingled with CAF-HI into NOD/SCID mice. The tumor growth pattern in NOD/SCID mice was similar to the one obtained in BALB/c mice: CAF-HI significantly enhanced tumor growth throughout the experiment and the tumor weight at the end of the experiment was significantly higher in the admixed group (Figure 8B). Because GFP expressed by inoculated CAF-HI can potentially elicit an immune response, NOD/SCID mice were used as recipients. CAF-HI-GFP enhanced tumor growth even in NOD/SCID mice (data not shown). Collectively, these data indicate that the host's immune system is not responsible for the enhanced tumor growth induced by CAF.

## Discussion

The mechanisms underlying hormone independent tumor growth are still unknown. In a recent study, we demonstrated that CAF-HI express higher levels of FGF-2 than CAF-HD. Moreover, we showed that in vitro, CAF-HI induced a significant increase in HD cell proliferation as compared to CAF-HD. In vivo, the exogenous inoculation of FGF-2 mimicked the effect of hormones inducing HD tumor growth [13]. In this study, we challenged the hypothesis that co-inoculated CAF-HI could bypass the hormone requirement of HD tumors. We found that inoculated CAF-HI did not persist within the tumor and thus, they failed to induce HD tumor growth. However, CAF-HI decreased tumor latency and increased angiogenesis in HI tumors.

CAF have recently been implicated in important aspects of epithelial solid tumor biology, such as progression, growth, angiogenesis and metastasis. Several studies have demonstrated that after tumor or cell transplantation the cancer cells recruit the stroma from the host. In 2004, using intravital microscopy, Duda et al [7] reported that the stroma from transplants of mammary cell lines was fully replaced by the host stroma after 4 weeks of transplantation. Two years later, Udagawa et al [21], using a lung cell line in GFP mice, demonstrated that this occurred several days after transplantation. Therefore, it is clear that once the tumor is established, the accompanying stroma is recruited from the host. However, in experiments regarding the inoculation of CAF together with tumor cells, the persistence of CAF within the tumor mass is not so well defined. Some studies have indicated that exogenously inoculated human CAF remain as part of the tumor stroma at least during the first 30 days [6] or more [5], and others have reported that inoculated human CAF disappear and the tumor stroma is exclusively of murine origin [4,8]. Here, we demonstrated that in our model, exogenously administered CAF-HI are undetectable as early as day 13 after injection. Thus, no long-lasting effects can be directly attributed to inoculated CAF. Despite their inability to persist, these CAF can exert growth promoting effects during the first days after cell inoculation decreasing tumor latency. The short lifespan of inoculated CAF-HI is not enough to enhance HD growth, although it rapidly creates a proper microenvironment for HI growth.

The fact that stromal components, such as CAF or Matrigel, increase the tumor burden is not a novel finding. In fact, this methodology is used to grow cells that will otherwise not grow [4]. Co-inoculation of fibroblasts with different tumor cells [4,5,22,23] or with sub-tumorigenic numbers of epithelial cells [24] has been shown to promote tumor growth. We found that co-inoculated CAF-HI enhanced HI tumor growth and that they do not persist within the tumor. Based on these findings, we concluded that the growth enhancing effects are a result of changes occurring during the early stages of tumor development. However, most studies using admixed CAF investigate their effects once the tumor is already settled and, according to our data, these CAF may be those recruited by the tumor cells [5,6].

To understand the stimulatory effects of CAF on tumor growth, we also investigated possible differences in the tumor site microenvironment at early time points after cell inoculation. We looked for changes occurring in the site of implantation even before the tumor structure was organized. In our view, the very first stages in tumor development are crucial and a fast stromal recruitment will favor tumor formation and progression. Both, growth promoting or inhibitory effects have been demonstrated for certain hematopoietic cells such as: mast cells, neutrophils, NK cells or dendritic cells [25,26]. Most of these studies using CAF and cell lines have been made using immunocompromised mice. The strength of this study is that we are working with BALB/c mice and we could not establish differences in the immune infiltrates that could account for the differences observed. Moreover, the same differences in tumor growth were observed in BALB/c mice and in NOD/SCID mice supporting a non-immune effect of CAF.

Accumulating evidence suggests a role for fibroblasts promoting angiogenesis [5,27]. Studebaker et al [28], have shown that MCF-7 cells co-injected with senescent skin fibroblasts, which secrete IL-6, developed tumors, while mice co-injected with pre-senescent skin fibroblasts, that express little or no IL-6, failed to form tumors. We previously demonstrated that CAF-HI secrete high levels of FGF-2 [13]. FGF-2 is a well known angiogenic factor [20] and here, we showed an increase in angiogenesis when EPI-HI were co-inoculated with CAF-HI. We can speculate that FGF-2 from CAF-HI might be contributing to the development of a pro-angiogenic milieu creating a permissive microenvironment that favors tumor growth.

The precise nature and origin of the stromal cells that grow along with the tumor cells is still a matter of debate. It has been proposed that CAF are resident fibroblasts recruited by the tumor [29], that they may arise by an epithelial mesenchymal transition (EMT) of the tumor parenchyma [30-32], or that they are mesenchymal bone marrow derived cells recruited from the circulation [21]. Although we cannot yet establish if CAF from our tumors are resident or bone marrow recruited fibroblasts, we can rule out the possibility of an EMT as CAF-HI isolated from tumors growing in BALB/c-GFP mice expressed GFP.

## Conclusions

We demonstrated that co-inoculated CAF-HI did not provide sufficient stimuli to replace the hormone requirement of HD epithelial cells in vivo. However, they facilitated HI epithelial growth at least in part by favoring angiogenesis. In addition, we demonstrated that the inoculated CAF are lost in a short period of time after injection and that tumor cells recruit fibroblasts from the host. The results suggest that careful controls should be included in experiments involving inoculated CAF to draw appropriate conclusions on the biology of cancer.

## Abbreviations

CAF: carcinoma associated fibroblasts; CAF-HD: hormone dependent carcinoma-associated fibroblasts; CAF-HI: hormone independent carcinoma-associated fibroblasts; CK: cytokeratin; EPI: epithelial cells; EPI-HD: hormone dependent epithelial cells; EPI-HI: hormone independent epithelial cells; ER: estrogen receptors; FISH: fluorescence *in situ *hybridization; GFP: green fluorescent protein; HD: hormone dependent; HI: hormone independent; MPA: medroxyprogesterone acetate; PI: propidium iodide; PMN: polymorphonuclear; PR: progesterone receptors; sc: subcutaneously; SMA: smooth muscle actin.

## Competing interests

The authors declare that they have no competing interests.

## Authors' contributions

CL and AM designed and conceived the manuscript; CAL carried out the transplantation experiments, drafted the manuscript and coordinated the manuscript; VTF performed the FISH analyses and intradermal injections. AS carried out the transplantation experiments, immunofluorescence and quantified tumor vessels. SV carried out the histological examinations. LC participated in the angiogenesis assays. All authors read and approved the final manuscript.

## Authors' Information

VTF, LC, CAL and CL: Members of the Research Career, CONICET. SIV: Staff pathologist, Academia Nacional de Medicina, Buenos Aires, Argentina. AM: Staff pathologist, OPCB, NIDCR, NIH, USA.

## Pre-publication history

The pre-publication history for this paper can be accessed here:

http://www.biomedcentral.com/1471-2407/10/293/prepub
